# Supplementary material for: In vivo recombination of Saccharomyces eubayanus maltose-transporter genes yields a chimeric transporter that enables maltotriose fermentation
Source: PLoS Genet. 2019 Apr 4;15(4):e1007853. doi: 10.1371/journal.pgen.1007853 (PMC6448828; doi:10.1371/journal.pgen.1007853)
Supplement: S1 Table — (DOCX) [file pgen.1007853.s001.docx]

**Table S1: Primers used in this study**

| **Primer #** | **Sequence 5' to 3'** | **Purpose** |
| --- | --- | --- |
| 4224 | TTGATGTAAATATCTAGGAAATACACTTG | ScSGA1 diagnostic out-out primer |
| 4226 | ACTCGTACAAGGTGCTTTTAACTTG | ScSGA1 diagnostic out-out primer |
| 5921 | AAAACTTAGATTAGATTGCTATGCTTTCTTTCTAATGAGC | p426 backbone amplification |
| 7812 | TCATGTAATTAGTTATGTCACGCTTACATTC | p426 backbone amplification |
| 8570 | GCGCTTTACATTCAGATCCCGAG | Diagnostic identification S. cerevisiae |
| 8571 | TAAGTTGGTTGTCAGCAAGATTG | Diagnostic identification S. cerevisiae |
| 8572 | GTCCCTGTAC CAATTTAATATTGCGC | Diagnostic identification S. eubayanus |
| 8573 | TTTCACATCT CTTAGTCTTTTCCAGACG | Diagnostic identification S. eubayanus |
| 9036 | TTTACAATATAGTGATAATCGTGGACTAGAGCAAGATTTCAAATAAGTAACAGCAGCAAACATAGCTTCAAAATGTTTCTACTCCTTTTTTAC | Fragment amplification for ScSGA1 integration with maltase |
| 9039 | CACCTTTCGAGAGGACGATGCCCGTGTCTAAATGATTCGACCAGCCTAAGAATGTTCAACGCCGCAAATTAAAGCCTTCG | Fragment amplification for ScSGA1 integration with maltase |
| 9355 | TGTAAATATCTAGGAAATACACTTGTGTATACTTCTCGCTTTTCTTTTATTTTTTTTTGTAGTTTATCATTATCAATACTCGCCATTTC | Maltase fragment amplification for ScSGA1 integration with transporter |
| 9596 | GTTGAACATTCTTAGGCTGGTCGAATCATTTAGACACGGGCATCGTCCTCTCGAAAGGTGGTGTGGAAGAACGATTACAACAG | Maltase fragment amplification for ScSGA1 integration with transporter |
| 10199 | TCCGTAGGTGAACCTGCGG | ITS1 forward |
| 10202 | TCCTCCGCTTATTGATATGC | ITS4 reverse |
| 10491 | GCTCATTAGAAAGAAAGCATAGCAATCTAATCTAAGTTTTAAAGTTTCGGTATACTTAGCAGACAG | MalT1 amplification with p426 backbone overhang |
| 10492 | GGAGGGCGTGAATGTAAGCGTGACATAACTAATTACATGATACCCTAATCAAGTAAATAGATAATAAAGTTAATGTG | MalT1 amplification with p426 backbone overhang |
| 10632 | GGAGGGCGTGAATGTAAGCGTGACATAACTAATTACATGATGCGCTAAGAGTCATCAAT | MalT2/4 amplification with p426 backbone overhang |
| 10633 | GCTCATTAGAAAGAAAGCATAGCAATCTAATCTAAGTTTTGAGGCGTGATATGCTCCAT | MalT2/4 amplification with p426 backbone overhang |
| 10671 | GGAGGGCGTGAATGTAAGCGTGACATAACTAATTACATGATGTCAGATAACAAAACCAGATACC | MalT3 amplification with p426 backbone overhang |
| 10672 | GCTCATTAGAAAGAAAGCATAGCAATCTAATCTAAGTTTTCGATAGAATATCCTGCTGAACC | MalT3 amplification with p426 backbone overhang |
| 11909 | ACTTGTTGGCTTCTCAAAGATGTC | Diagnostic identification S. eubayanus |
| 12635 | CACGAACCATGTCCGTGTAG | SeSGA1 diagnostic out-out primer |
| 12636 | GTTGGACGTTCCGGCATAGC | SeSGA1 diagnostic out-out primer |
| 13559 | GCCCTGAAAGCCGTTATCCATTTCGTTGTTACACAAGAAGATTTGCAGCGCCAGGACCCACATAGCTTCAAAATGTTTCTACTCCTTTTTTAC | Fragment amplification for SeSGA1 integration |
| 13560 | TTCTTGTCTTATTTGATGGGCGTCCCAAAATGAGGTGTAGGACCAAGTGAGGTGCCGAGCGCAAATTAAAGCCTTCGAGCG | Fragment amplification for SeSGA1 integration |
