# Supplementary figures and images for: In vivo recombination of Saccharomyces eubayanus maltose-transporter genes yields a chimeric transporter that enables maltotriose fermentation
Source: PLoS Genet. 2019 Apr 4;15(4):e1007853. doi: 10.1371/journal.pgen.1007853 (PMC6448828; doi:10.1371/journal.pgen.1007853)

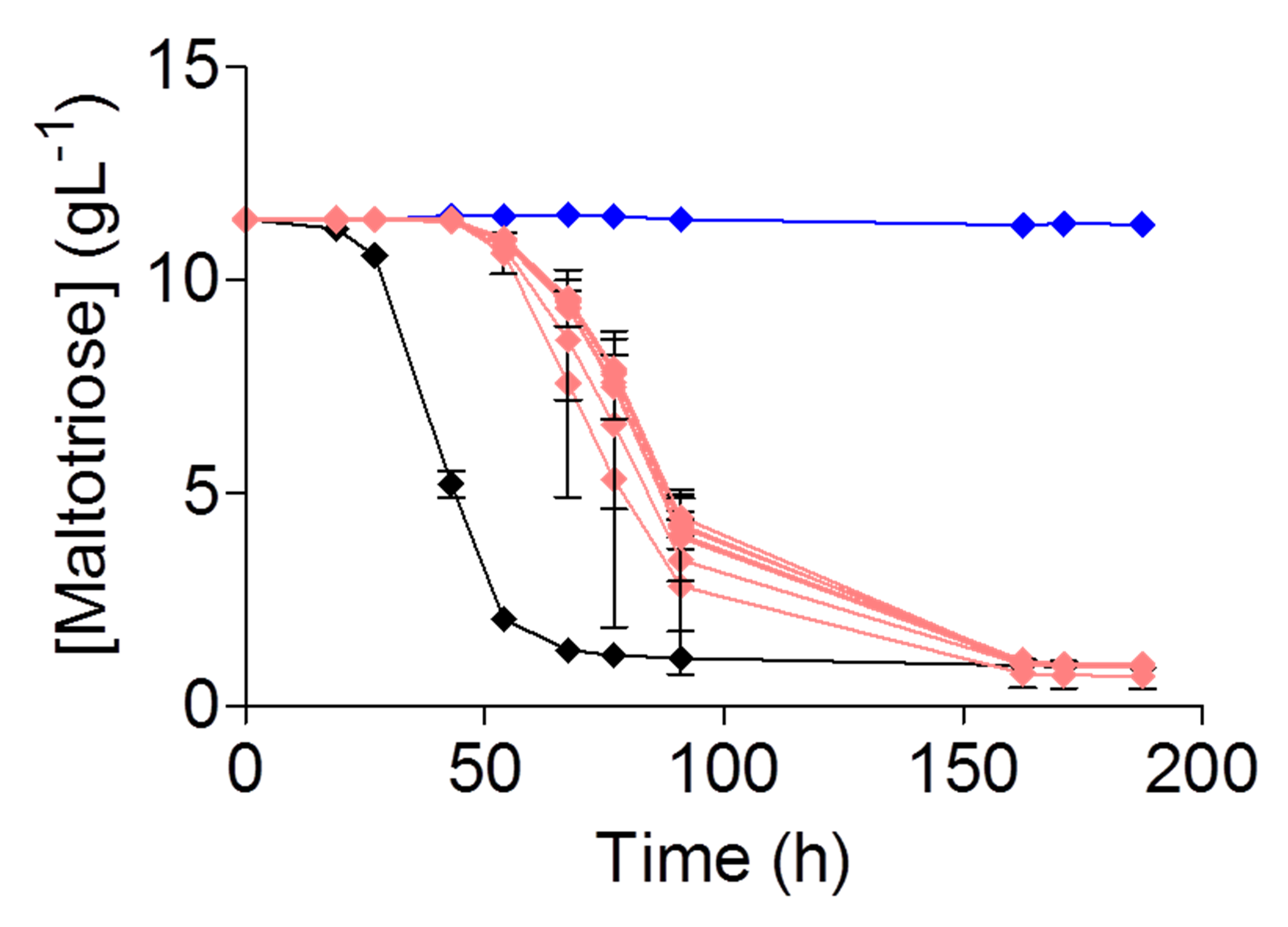

Supplement: S1 Fig — Characterization of S. pastorianus CBS 1483 (black), S. eubayanus CBS 12357T (blue) and selected mutants IMS0637-IMS0643 (light red) on SMMt at 20°C. The average concentration of maltotriose (diamonds) and average deviation were determined from two replicates. IMS0637 was chosen as representative for all mutants (S8 Data File). (TIF) [file pgen.1007853.s002.TIF]

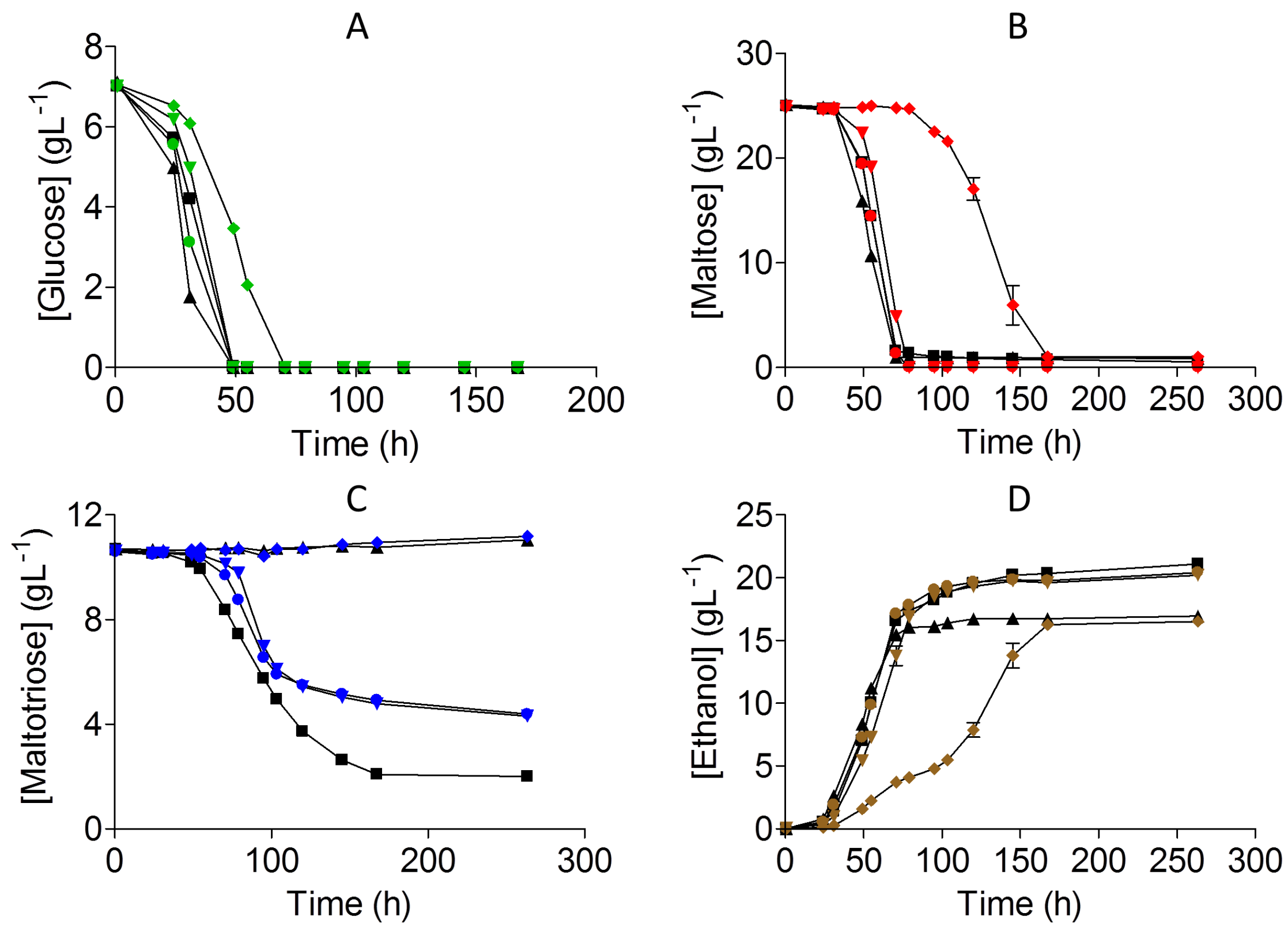

Supplement: S2 Fig — (A) Consumption of glucose, (B) maltose, (C) maltotriose and (D) ethanol were measured by HPLC. Data represent average and standard deviation of three biological replicates (S9 Data File). (TIF) [file pgen.1007853.s003.TIF]

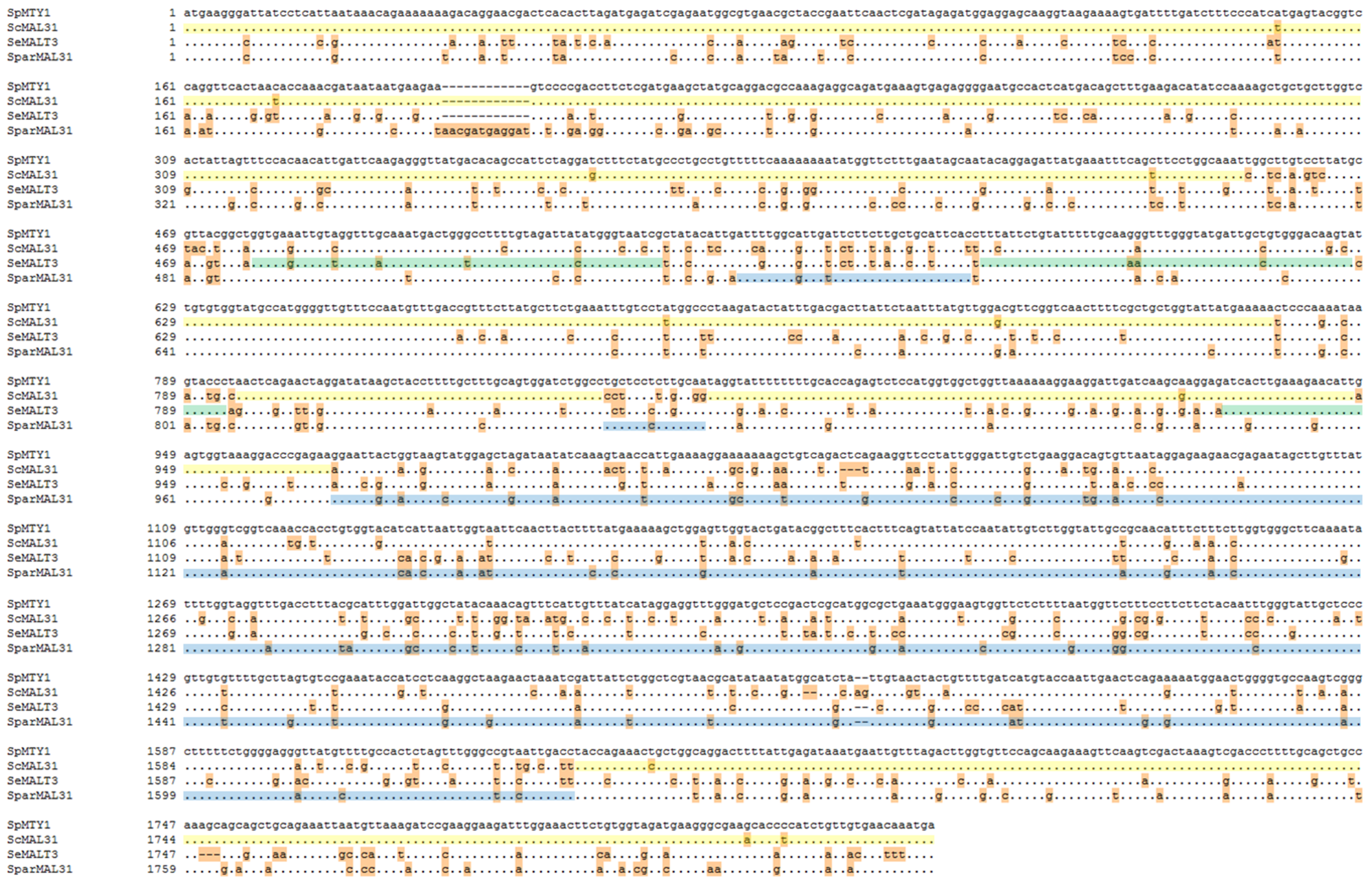

Supplement: S3 Fig — The allignment was performed using the Clone manager software (version 9.51, Sci-Ed Software). Identical nucleotides are shown by dots and nucleotides which differ from SpMTY1 are shown in orange. In addition, the sequences which match exactly are highlighted in yellow for SeMALT4, in green for SeMALT1 and in blue for SeMALT3. (TIF) [file pgen.1007853.s004.TIF]

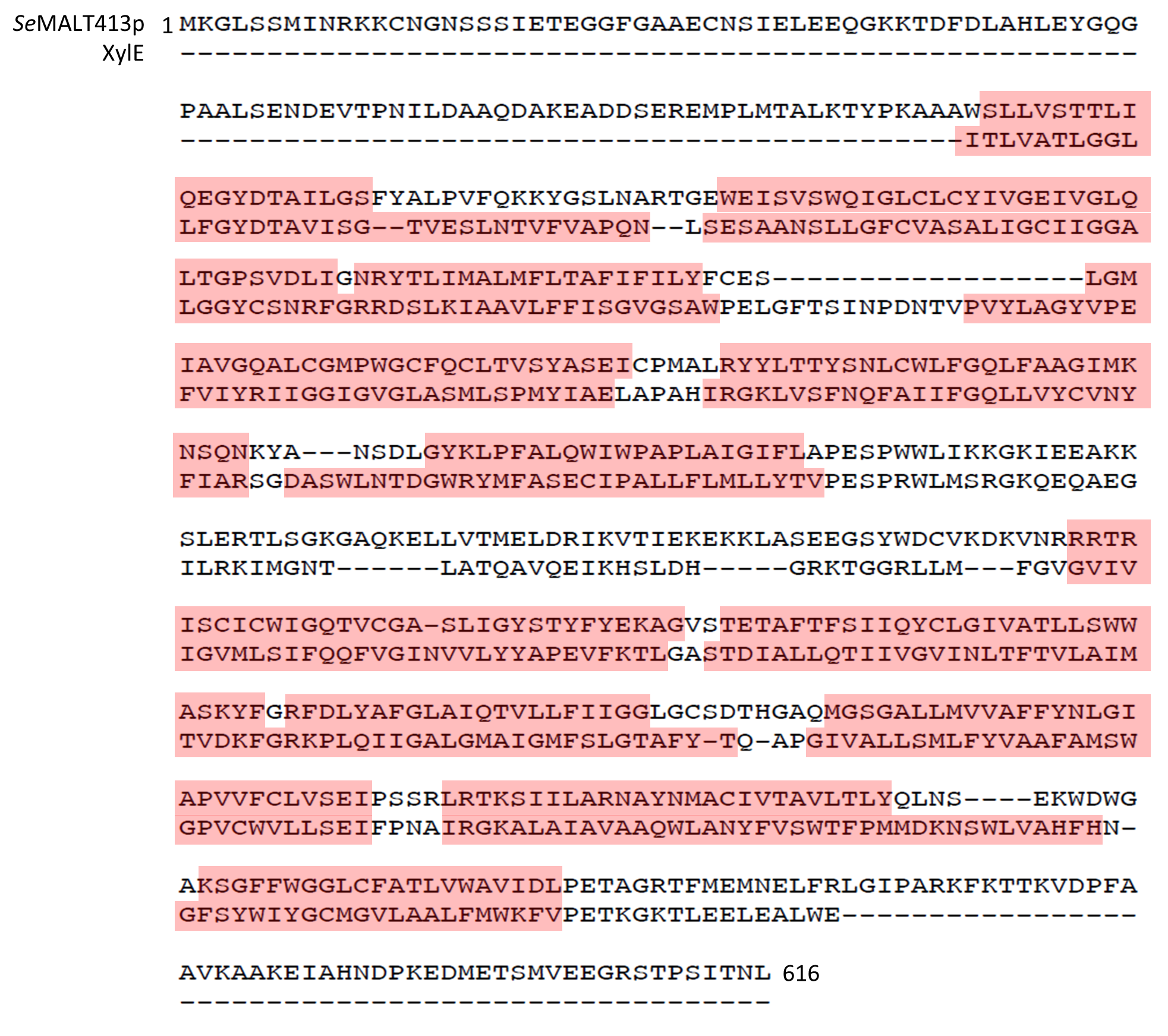

Supplement: S4 Fig — Transmembrane domain α-helices are indicated in red. (TIF) [file pgen.1007853.s005.TIF]

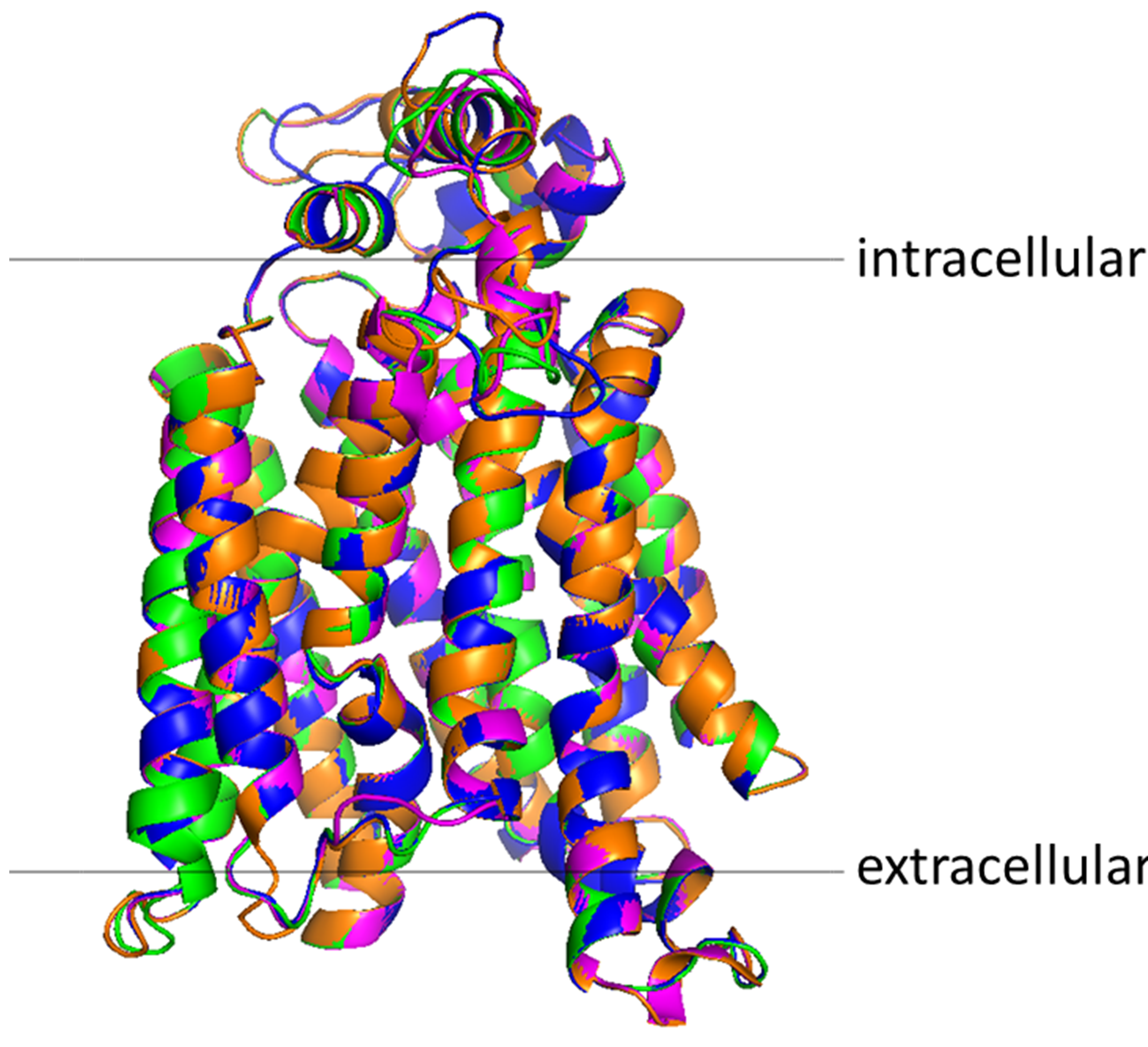

Supplement: S5 Fig — SeMALT genes were translated into amino acid sequence and used for structural prediction using SWISS-MODEL with XylE as a structural template. Resulting SeMalt protein structures were overlayed using PyMOL. (TIF) [file pgen.1007853.s006.TIF]

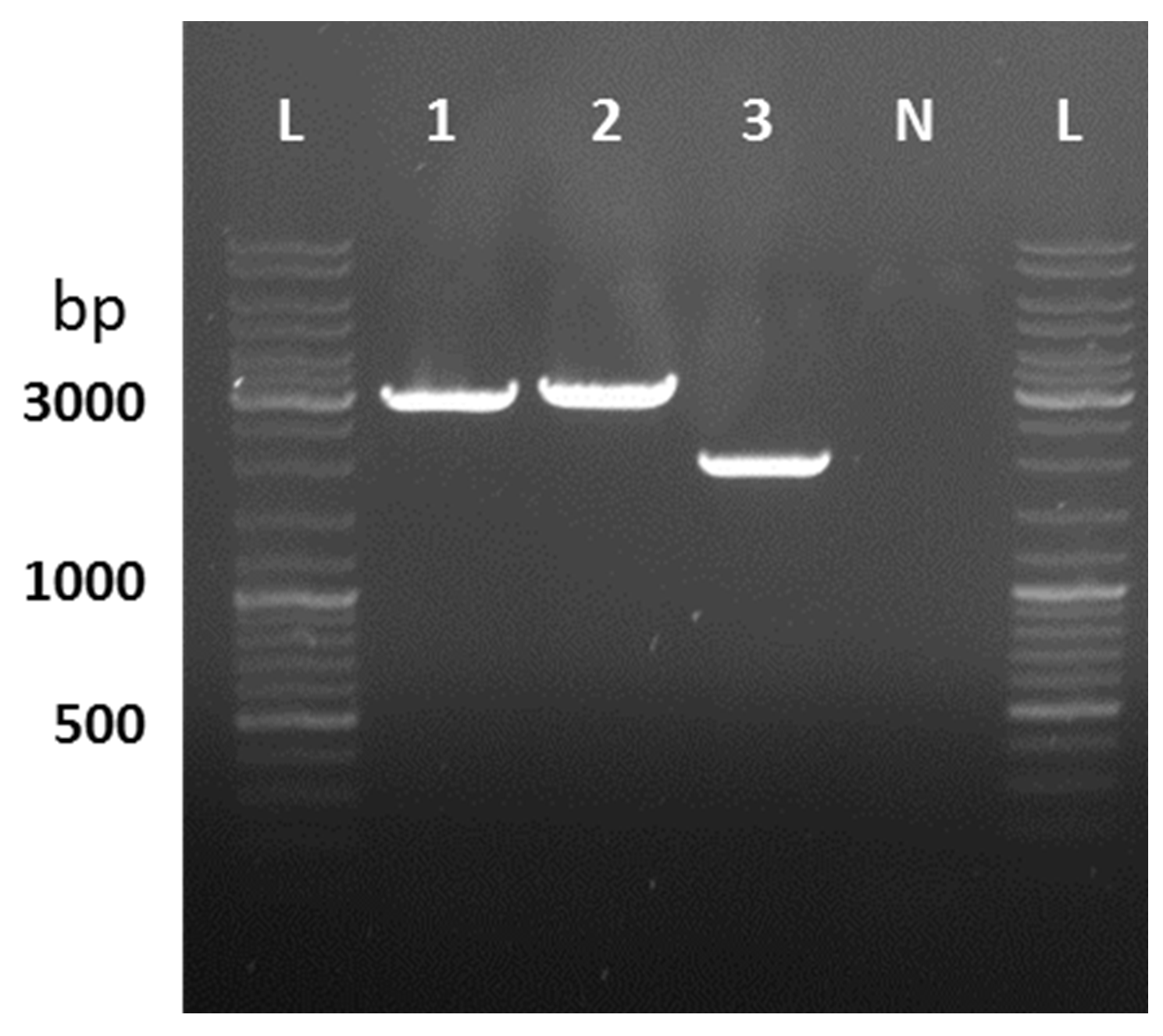

Supplement: S6 Fig — The SeSGA1 locus was amplified from genomic DNA of IMX1941 (1), IMX1942 (2) and CBS 12357T (3) using primers 12635/12636 and Phusion polymerase (Thermo Fischer Scientific). At the SeSGA1 locus, IMX1941 should harbor ScTEF1p-SeMALT2-ScCYC1t and IMX1942 should harbor ScTEF1p-SeMALT413-ScCYC1t. As a negative control, a PCR was done with primers 12635/12636 without template DNA. L indicates the GeneRuler DNA Ladder Mix (Thermo Fischer Scientific). (TIF) [file pgen.1007853.s007.TIF]

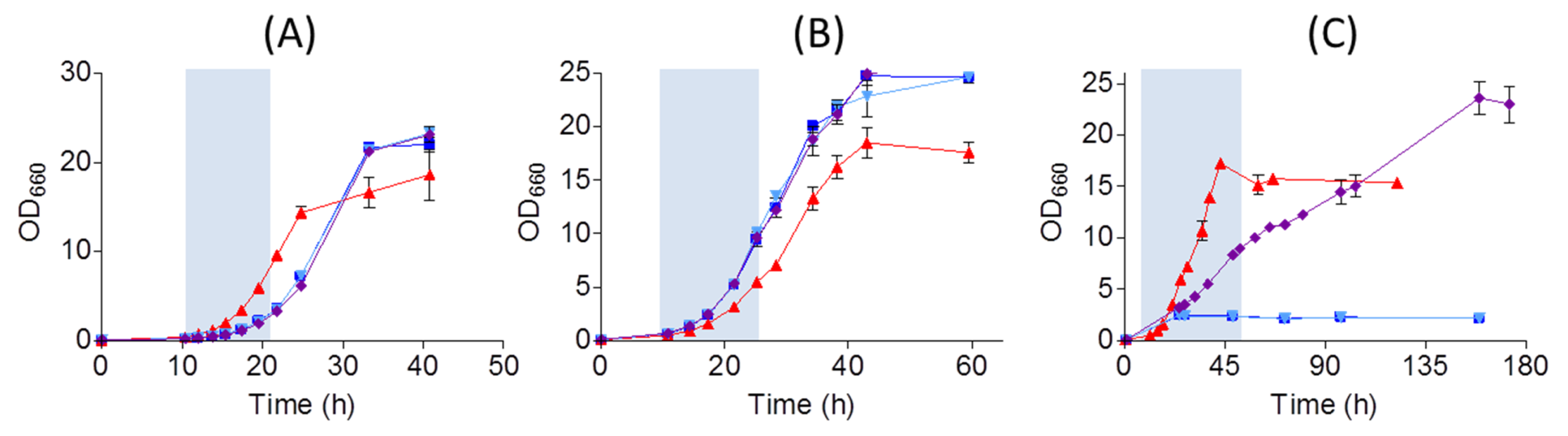

Supplement: S7 Fig — Characterization of CBS 12357T (blue squares), IMS0750 (red triangles), IMX1941 (cyan triangles), IMX1942 (purple diamonds) on SM (A) glucose, (B) maltose and (C) maltotriose. Strains were cultivated at 20°C and optical densities were measured at 660 nm. Data represent average and standard deviation of three biological replicates (S10 Data File). Blue boxes represent the timeframe used to calculate growth rates. (TIF) [file pgen.1007853.s008.TIF]

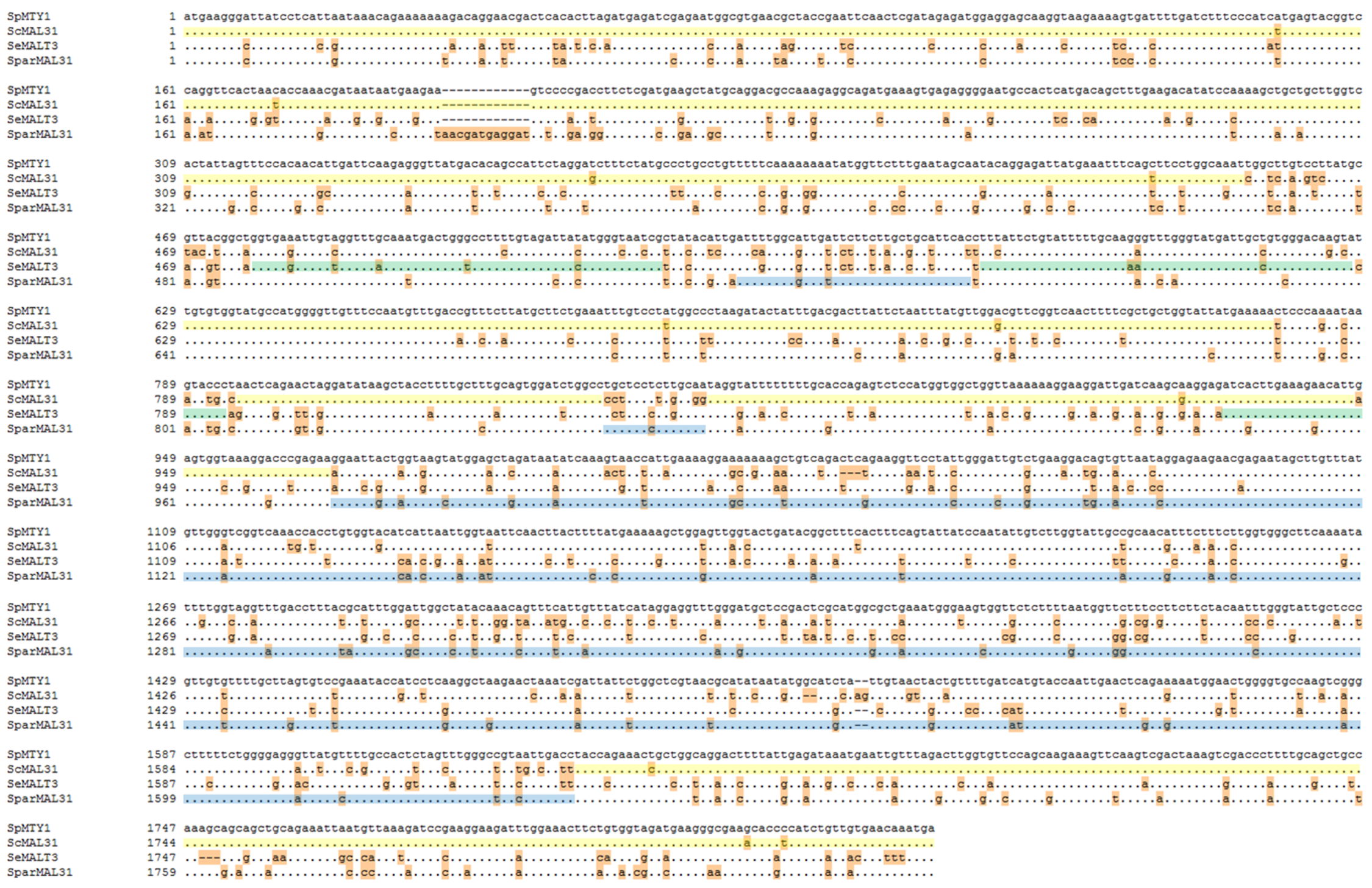

Supplement: S8 Fig — Identical nucleotides are shown by dots and nucleotides which differ from SpMTY1 are shown in orange. In addition, the high-identity sequences described in the main text are highlighted in yellow for ScMAL31, in green for SeMALT3 and in blue for SparMAL31. (TIF) [file pgen.1007853.s009.TIF]

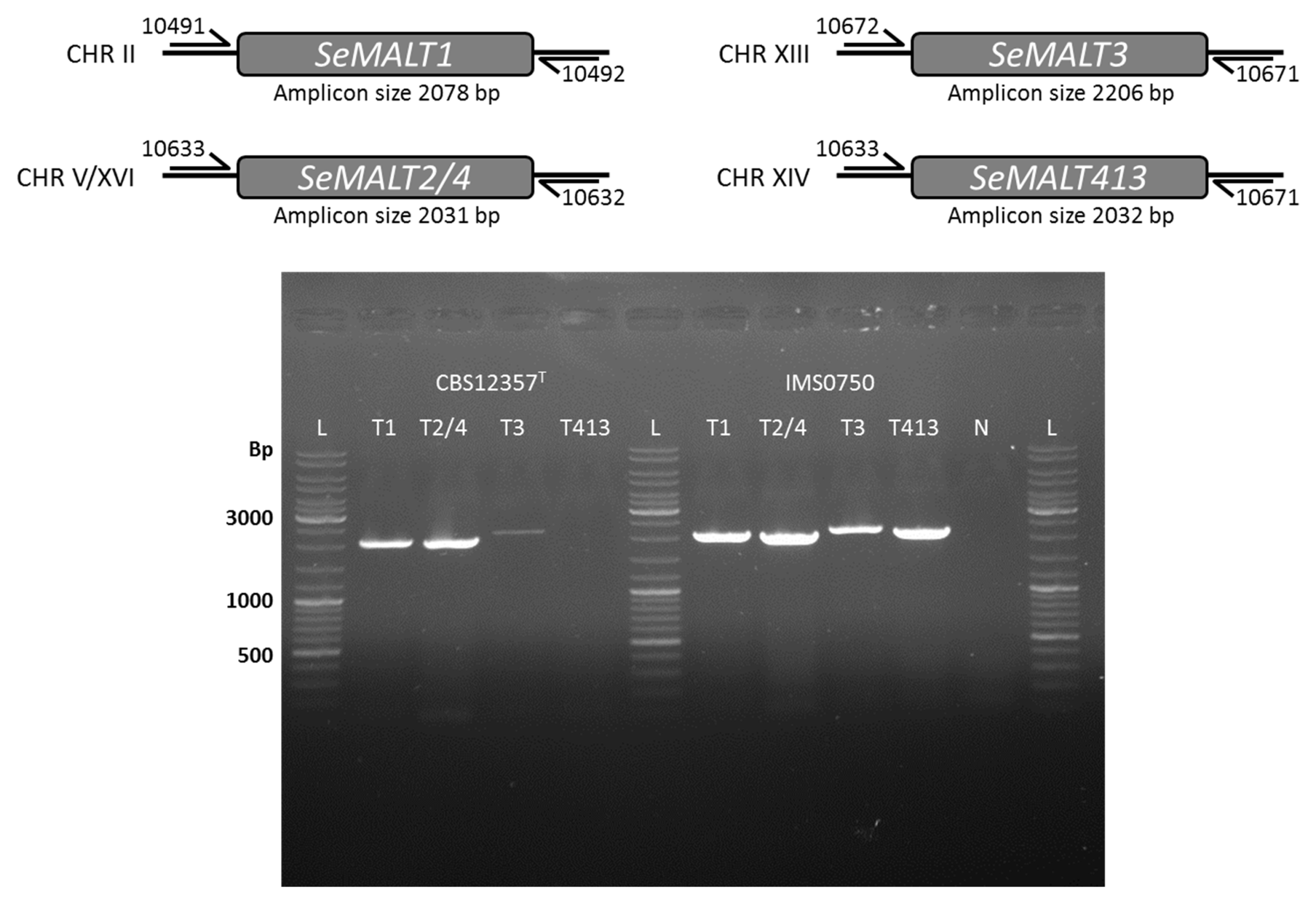

Supplement: S9 Fig — The SeMALT genes were amplified from genomic DNA of CBS 12357T and IMS0750 using Phusion polymerase (Thermo Fischer Scientific). Lanes show PCR products for SeMALT1 (primers 10491/10492), SeMALT2 and SeMALT4 (primers 10633/10632), SeMALT3 (primers 10672/10671) and SeMALT413 (primers 10633/10671). As a negative control, a PCR was done with primers 10633/10632 without template DNA. L indicates the GeneRuler DNA Ladder Mix (Thermo Fischer Scientific). (TIF) [file pgen.1007853.s010.TIF]
